# Dr. F. H. Badger's Method of Filling Cavities in Posterior Approximal Surfaces of Molar Teeth

**Published:** 1856-01

**Authors:** 


*Dr. F. H. Badger's Method of Filling Cavities in Posterior Approximal Surfaces of Molar Teeth.*—As this method, adopted some years since, of filling cavities in the posterior approximal surface of molar teeth when difficult of access, has recently been discussed by some of the members of the profession, it gives us pleasure to be able to present, in the present number of the Journal, a brief description of it furnished by Dr. Badger. As a most skillful and finished operator, Dr. B. has been known to most of the older members of the profession in the United States for more than a quarter of a century, and any thing from the pen of so old and eminent a practitioner, will, we are well assured, be read with pleasure. We would be glad if the doctor would continue to furnish contributions to our pages from the ample storehouse of his large experience.

